# Urinary kynurenine,tryptophan, and neopterin concentrations during physiological pregnancy

**DOI:** 10.1038/s41598-025-04642-9

**Published:** 2025-06-04

**Authors:** Lenka Kujovská Krčmová, Kateřina Matoušová, Lenka Javorská, Kristýna Mrštná, Nikola Přívratská, Chaweewan Suwanvecho, Dorota Turoňová, Mohamed Farrag, Marian Kacerovský, Bohuslav Melichar

**Affiliations:** 1 Department of Clinical Biochemistry and Diagnostics, University Hospital Hradec Králové, Sokolská 581, 500 05 Hradec Králové, Czech Republic; 2 Department of Analytical Chemistry, Faculty of Pharmacy in Hradec Králové, Charles University, Akademika Heyrovského 1203/8, 500 05 Hradec Králové, Czech Republic; 3 Gynecology Clinics Hradec Králové, Malé náměstí 7, 500 03 Hradec Králové, Czech Republic; 4 Department of Obstetrics and Gynecology, University Hospital Olomouc, I.P. Pavlova 6, 779 00 Olomouc, Czech Republic; 5 Department of Oncology, Faculty of Medicine and Dentistry, Palacký University, Olomouc, I.P. Pavlova 6, 779 00 Olomouc, Czech Republic

**Keywords:** Urinary kynurenine, Urinary tryptophan, Healthy pregnancy, Stability study, HPLC-MS/MS

## Abstract

During pregnancy the alterations of kynurenine and tryptophan metabolism play an important role in local signalling and the prevention of fetal rejection. The aim was to investigate urinary levels of kynurenine and tryptophan during physiological pregnancy, and to determine their stability in urine during sample storage at different conditions. Urinary samples were obtained from 73 healthy pregnant women (median age 31 years), during the 1st, 2nd, and 3rd trimesters and from 42 healthy non-pregnant women (median age 30 years). Liquid chromatography methods using selective and sensitive mass spectrometry detection were used for analysis. Urinary neopterin, kynurenine, and tryptophan increased during the pregnancy and in comparison to the non-pregnant women. No correlation of the analytes with gestation age within each trimester and among the different analytes was observed. Kynurenine and tryptophan were stable in urine for 14 days at 4 °C, 6 months at − 22 °C, and 12 months at − 84 °C. Present results demonstrate differences in urinary concentrations of kynurenine, tryptophan, and neopterin between women with physiological pregnancy and healthy women. Simultaneous determination of kynurenine, tryptophan and neopterin may be explored in the disorders of pregnancy in future investigations.

**Supplementary Information:**

The online version contains supplementary material available at 10.1038/s41598-025-04642-9.

## Introduction

The essential amino acid tryptophan plays an important role in the regulation of the diurnal cycle, immune response, protein biosynthesis and serves as precursor of melatonin, serotonin, and vitamin B3.^1,2^.

Tryptophan is mostly (over 90%) metabolized by indoleamine 2,3-dioxygenase or tryptophan 2,3-dioxygenase to N-formylkynurenine which is rapidly hydrolyzed to kynurenine. Individual metabolites have many important anti-inflammatory, anti-oxidative, and neuroprotective functions in the nervous, endocrine and immune systems, and play an important role in many inflammatory disorders^3,4^. Disorders affecting the kynurenine pathway can manifest as primary (genetic) or secondary (due to inflammatory response) and usually result in dysregulation of the key enzymes and further accumulation of some toxic metabolites (e.g. neurotoxic quinolinic acid) or disbalance in the entire pathway. Abnormal activation of the tryptophan and kynurenine pathway can contribute to the development of osteoporosis, polycystic ovary syndrome, inflammatory bowel disease, cardiovascular disease, or other conditions^5^.

Neopterin is a pteridine metabolite of guanosine triphosphate used as a biomarker of cellular immune system activation. Neopterin is produced by macrophages and dendritic cells after stimulation with interferon gamma^6^. High concentrations of neopterin have been reported in patients with viral or bacterial infections, including hepatitis B and C viruses, human immunodeficiency virus, or tuberculosis, and autoimmune or neoplastic disorders^7,8^. Neopterin is a useful parameter for monitoring disease prognosis and therapy and may serve as a predictor of outcome in patients with cardiovascular disorders, viral infection, and cancer.

**Fig. 1:**
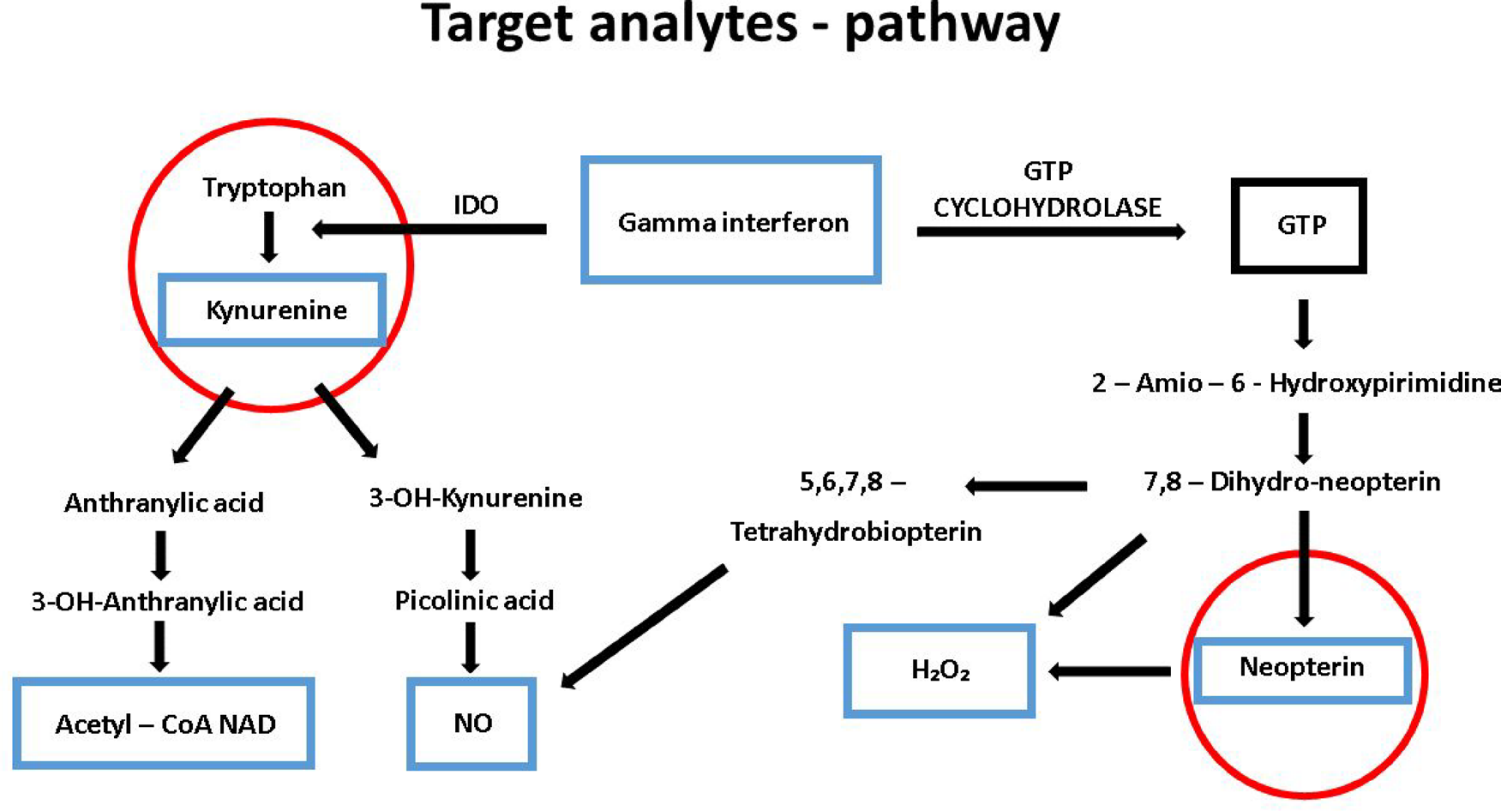
Biochemical pathway of kynurenine and tryptophan production after stimulation of interferon gamma (GTP, guanosine triphosphate; IDO, indoleamine 2,3-dioxygenase; NO, nitric oxide; Acetyl-CoA NAD, acetyl coenzyme A nicotinamide adenine dinucleotide).

Neopterin and kynurenine concentrations usually correlate, which is based on their association with interferon gamma stimulation during inflammation. (Fig. 1). Each analyte demonstrates different part of the biochemical pathway during inflammation. Therefore the simultaneous determination of neopterin and kynurenine/tryptophan is advantageous and was also used in many clinical investigations evaluating patient prognosis or for monitoring of therapeutic interventions^9–11^.

Recently, several studies have explored tryptophan metabolism during pregnancy, kynurenine/tryptophan and neopterin concentrations as biomarkers in maternal or umbilical cord blood^12–15^. Most of these methodologies use liquid chromatography with various types of detection such as fluorescence or mass spectrometry. The possibilities of determining kynurenine in different biological matrices have been described in detail in the review Mrstna K. et al.^16^.

It has been demonstrated that the kynurenine pathway has a pivotal role in physiological conception in the prevention of fetal rejection^17^. The placental kynurenine synthesis has important roles as a local signaling pathway, providing a source of de novo NAD + synthesis, and regulating tryptophan and kynurenine metabolite supply to the growing fetus. Imbalance or dysfunction in the kynurenine pathway can lead to immune and vascular impairments and los of tolerance. It was reported that indoleamine 2,3-dioxygenase is highly expressed in the placenta and reduced in placentas from complicated pregnancies. It is believed that tryptophan 2,3-dioxygenase may contribute to feto-maternal tolerance and could compensate for placental immunosuppression when indoleamine 2,3-dioxygenase is absent. Tryptophan 2,3-dioxygenase also plays a role in angiogenesis or vessel tonicity^17,18^.

Serum neopterin was also monitored in asymptomatic pregnant women and was shown to predict preterm birth in the study reported by Navolan et al.^19^.

Serum concentrations of neopterin, sialic acid, and C-reactive protein were also studied in preeclampsia, and only neopterin levels were found elevated indicating an association between inflammation and cellular immune activation during preeclampsia^20^. Serum neopterin concentrations were reported to be associated with preterm birth as opposed to other inflammatory markers such as C-reactive protein^21^.

The kynurenine/tryptophan pathway was also studied in pregnancy complications. It was reported, that serum tryptophan concentrations were lower in pregnant women with preterm delivery compared to women with uneventful pregnancy. This decrease was explained as a consequence of inflammation or changes in energy metabolism^22^. Prescott et all reported in 2025 that tryptophan and its metabolites have potential as biomarkers for different pregnancy-related outcomes and further research should explore the mechanisms by which TRP metabolism impacts maternal and fetal health^23^.

Another study Fuchs et al. reported decreased plasma tryptophan concentrations during the 3rd trimester in comparison to earlier during the pregnancy and in healthy non-pregnant women, possibly due to enhanced tryptophan degradation during pregnancy as a result of cellular immune activation^24^.

All reported studies are based on the determination of neopterin, kynurenine and tryptophan in blood or amniotic fluid.

Tryptophan metabolism was also investigated in pregnant women with gestation diabetes levels of tryptophan were studied using 20 samples of amniotic fluid^25^. Another study is dealing with tryptophan levels in blood and urine but one-time only in 12.-26. week of pregnancy^26^. This amino acid was included in metabolome study in 1035 pregnant women, but 6.-9. week postpartum^27^. These studies didn’t investigate kynurenine levels. Tryptophan metabolic pathway was investigated by Law et al. in 2017 in urine of pregnant women with gestation diabetes mellitus. Levels of tryptophan and kynurenine were not reported^28^.

The use of urine as a sample matrix may be seen as an advantage in several aspects. The principal advantages are the non-invasive collection which does not burden the patient, in sufficient quantity of different assays, easy handling, and stability. Differences in urine dilution can be easily corrected by calculating the ratio to creatinine^29,30^.

To the best of our knowledge, there are no reports on urinary levels of kynurenine and tryptophan during pregnancy.

This study aimed to determine urinary kynurenine and tryptophan concentrations in each trimester of the low risk-uncomplicated pregnancy and explore the variation in each trimester. One of the most important goals of this study was to find a potential correlation between gestational age because most of the parameters/ biomarkers evaluated in pregnancy are related to gestational age. We were also interested in urinary neopterin concentrations, which were already studied in pregnancy, and their possible correlation with kynurenine/tryptophan levels. This study also examines the view of perspective using these analytes as biomarkers of pathological conditions associated with cellular immune activation or defect in tryptophan metabolism during pregnancy.

Second aim was to explore urinary samples stability during various conditions of storage for determination of kynurenine and tryptophan, which is important for future research, clinical studies and routine measurement.

## Patients and methods

Samples were obtained from 73 heathy pregnant women with low-risk singleton pregnancies without any pregnancy-related and/or chronic diseases, median age 31 (range 21–47) years specification of the studied group is in Table 1. Samples were collected from December 2020 to February 2022. The study protocol was approved by the institutional ethics committee of University Hospital Hradec Kralove (No 202011 P05), and all women signed informed consent. Gestational age was calculated based on fetal biometry (crown-rump length) in the first trimester. Gestation age at the time of sample collection (median and range) in the 1st, 2nd and 3rd trimester was 78 (73–87) days,120 (88–135) days, and 232 (216–262) days, respectively.

**Table 1:** Specification of the studied group.

Primiparous	56.5%
Body mas index 1st trimester median (range)	22.77 (16.83–39.52)
Body mas index 2nd trimester median (range)	23.80 (17.72–40.65)
Body mas index 3rd trimester median (range)	27.23 (19.49–44.41)
Newborn gender (n = 67)*	39 males / 28 female
Newborn weight (g) median (range)	3310 (2400–4360)
Gestational age at delivery (weeks + days) median (range)	39 + 4 (37 + 0–41 + 5)

*No information about newborn gender in 6 cases.

The group of non-pregnant women consisted of 42 women, median age 30 (range 18–47) years, who signed institutional ethics committee approved informed consent (No 202007 S01P), and all women signed informed consent. Samples were collected from August 2020 to September 2022.

The ethical committee approved the research and we confirm that all research was performed in accordance with relevant guidelines/regulations of the institution. Informed consent was obtained from all participants, all subjects and/or their legal guardian(s). Research was performed in accordance with the Declaration of Helsinki.

Urinary neopterin and creatinine were determined using HPLC-FLD/PDA method^10^. Briefly, 100 µL urine was centrifuged for 45 s at 14,100 × g, diluted 1:10 with 15 mM phosphate buffer at pH 6.5, and 170 µL is filtered using microtiter plates with 0.2 μm pore size filters and injected into the HPLC system. Analysis was performed using monolithic column High Resolution C18 150 × 4.6 mm and 15 mM phosphate buffer (pH 6.5) as the mobile phase. Neopterin was detected using fluorescence detection at 353 nm excitation and 438 nm emission wavelength, creatinine using photodiode-array detection using 235 nm.

Kynurenine and tryptophan were measured by UHPLC-MS/MS system using chromatographic column Kinetex Polar C18 100 × 4.6 mm, 2.6 µm protected with security guard column EVO C18 3 mm ID (Phenomenex, USA). The mobile phase was composed of 65% 5 mmoL/L ammonium formate buffer (solvent A) and 35% methanol with 0.2% formic acid (solvent B) with the flow rate 0.6 mL/min. Samples were proceed using 200 µL of urine add 10 µL of internal standard (1500 µmol⋅L ^ −1^ L-tryptophan-D5), then diluted by 800 µL solvent (1:1 A:B mobile phase). Samples were vortexed, then centrifuged (14 000 × g, 5 min) and filtrate through filtration plate with 0.2 μm pore size filters.

5 µL of sample were injected into LCMS system. Compounds were detected using a Shimadzu LCMS 8050 triple quadrupole mass spectrometer (Kyoto, Japan) in positive electrospray ionization mode.

For quantitation was used total ion current which represents the combined intensity of all detected masses at each point during the analysis.

For each analyte were used different mass transitions (specified in Table S1). Desolvatation line temperature was set as 250 °C, nebulizer gas flow 3 L⋅min^ −1^, heating block temperature as 400 °C, and drying gas flow 10 L⋅min^ −1^ All parameters were optimized by using the LabSolution automated optimization software. System operation, data acquisition, and data processing were controlled by the LabSolutions 5.188 software.

All methods were validated using the European Medicine Agency and the US Food and Drug Administration guidelines^31,32^.

Stability of urine samples for the determination of kynurenine and tryptophan was investigated using three different levels of real samples. Each sample was proceeded and measured three times using the same methods as in the study group. Stability was calculated as ratio stability in time/stability at day 0. The concentrations of target analytes in day 0 were considered as 100% and further results were related to this value the samples were evaluated as stable while the ratio was ± 10%. Data were also evaluated by the relative standard deviation method (including sample preparation and analysis), which is a statistical tool used to measure the variability or precision of a dataset relative to its mean to record any errors that may have occurred during the sample preparation and measurements.

Concentrations of all analytes were calculated as ratios to creatinine that was determined simultaneously with neopterin to correct for urine dilution or reported as kynurenine/tryptophan ratio.

The statistical analysis including correlation analysis (Spearman correlation), Mann–Whitney test, and Wilcoxon test for paired analysis was performed using the NCSS software (Kaysville, UT, USA). A p-value of 0.05 or below was considered statistically significant. Group size with statistical power ≥ 0.8 was n = 60 for pregnant women and n = 40 for non-pregnant women (except in comparison 1st trimester of pregnant women and non-pregnant women in kynurenine/tryptophan levels).

## Results

**Fig. 2:**
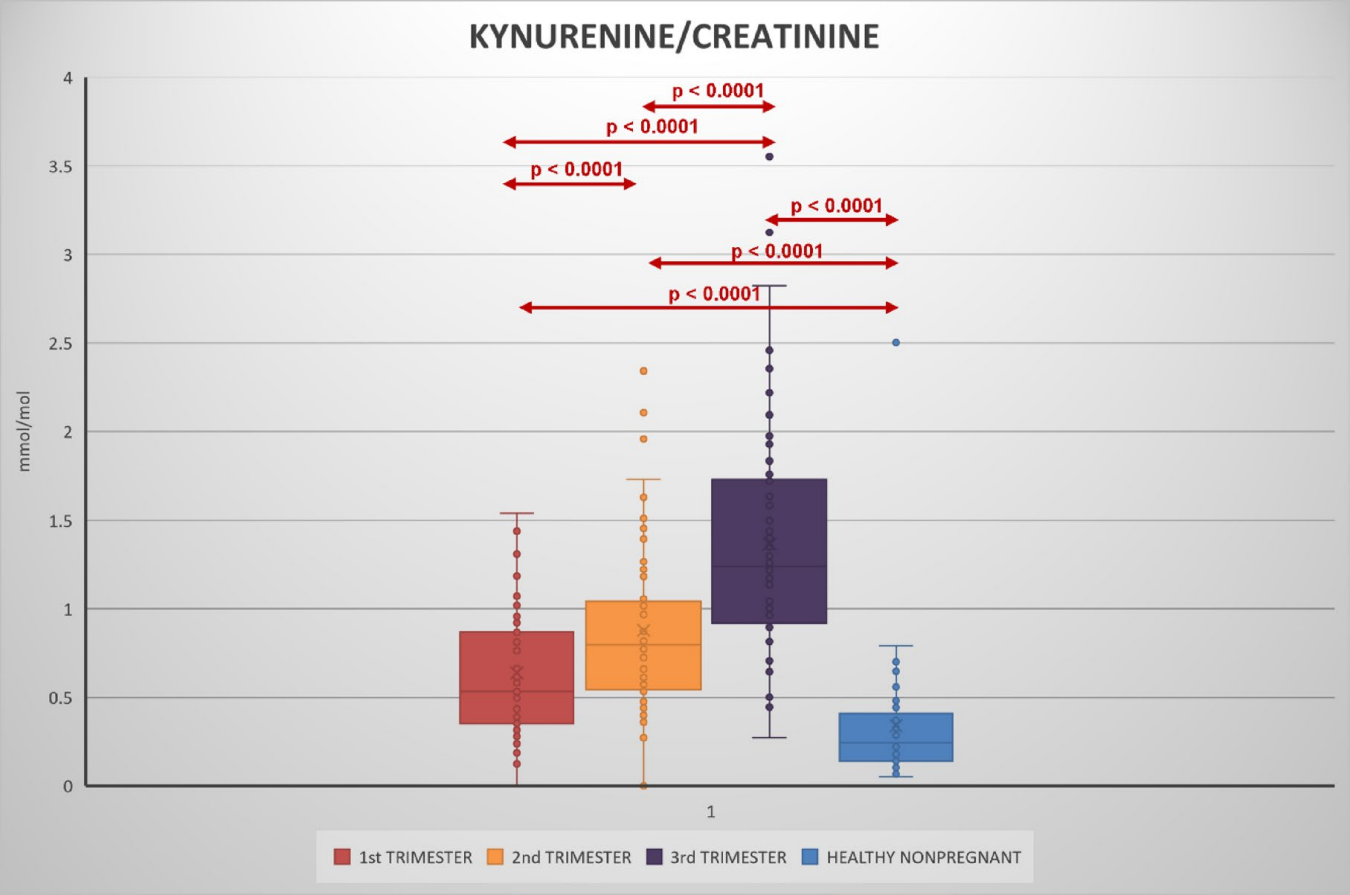
Urinary kynurenine/creatinine levels in 1st, 2nd, and 3rd trimester of pregnancy and its comparison during pregnancy and with healthy non-pregnant women.

**Fig. 3:**
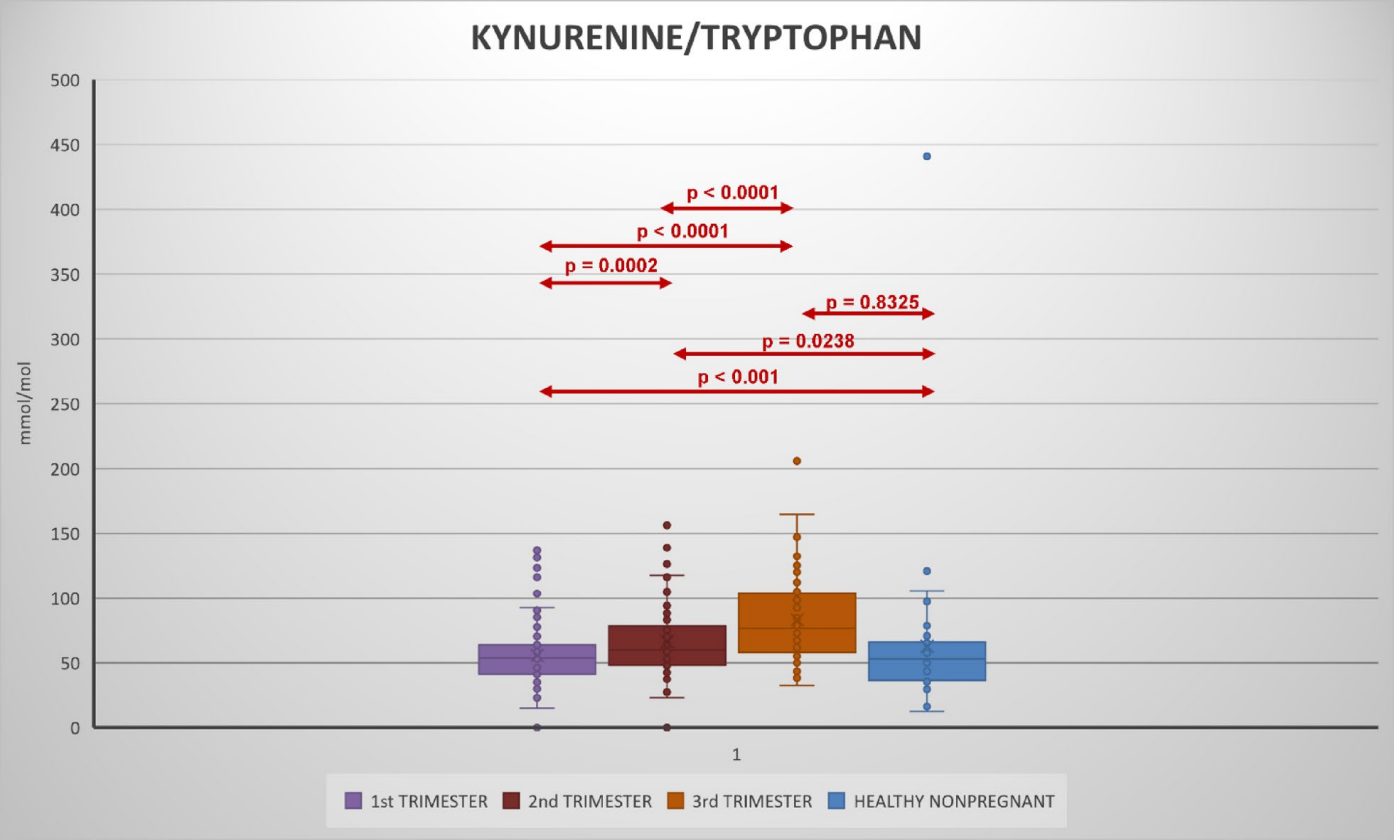
Urinary kynurenine/tryptophan levels in 1st, 2nd, and 3rd trimester of pregnancy and its comparison during pregnancy and with healthy non-pregnant women.

**Fig. 4:**
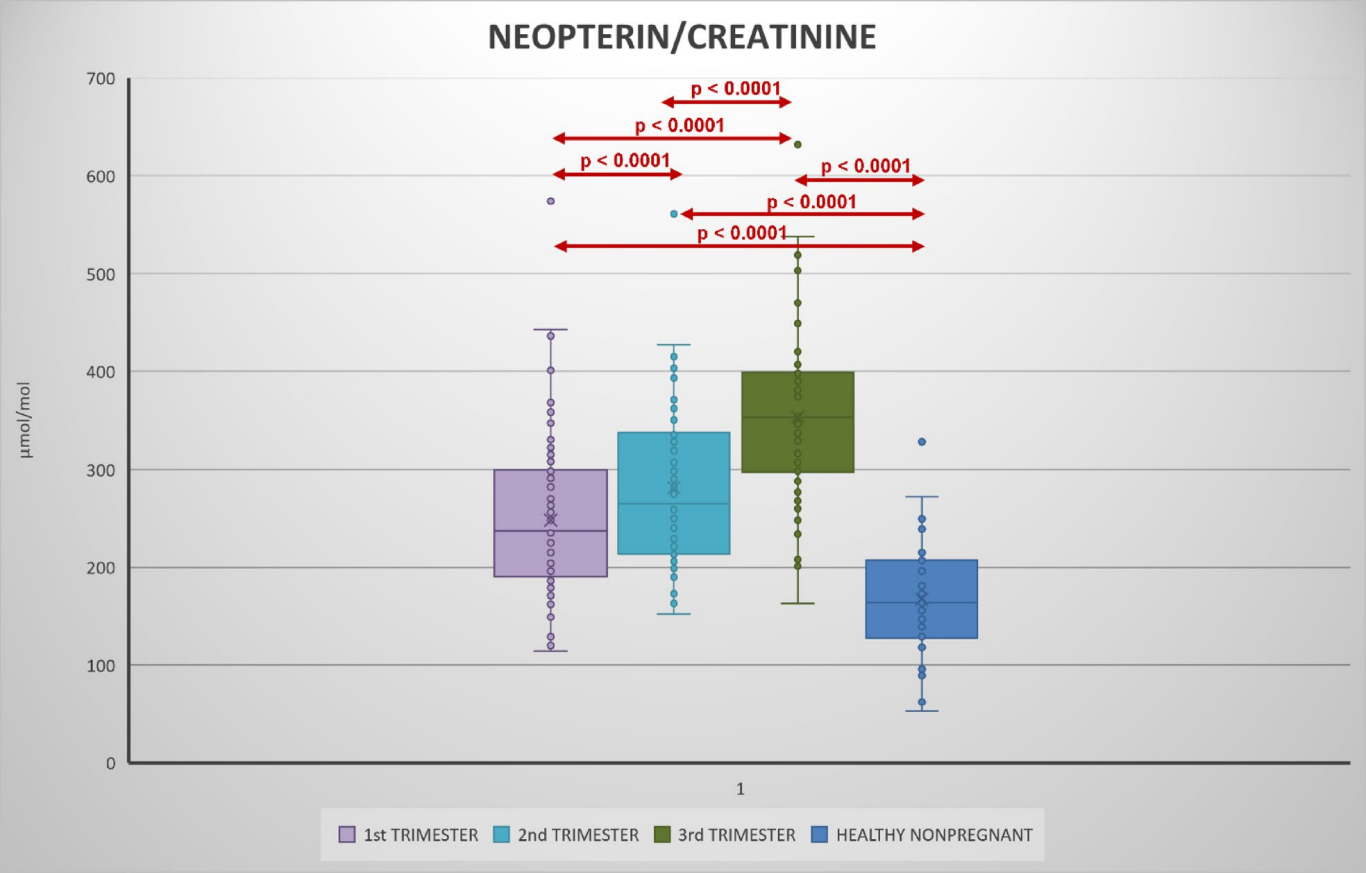
Urinary neopterin/creatinine levels in 1st, 2nd, and 3rd trimester of pregnancy and its comparison during pregnancy and with healthy non-pregnant women.

**Fig. 5:**
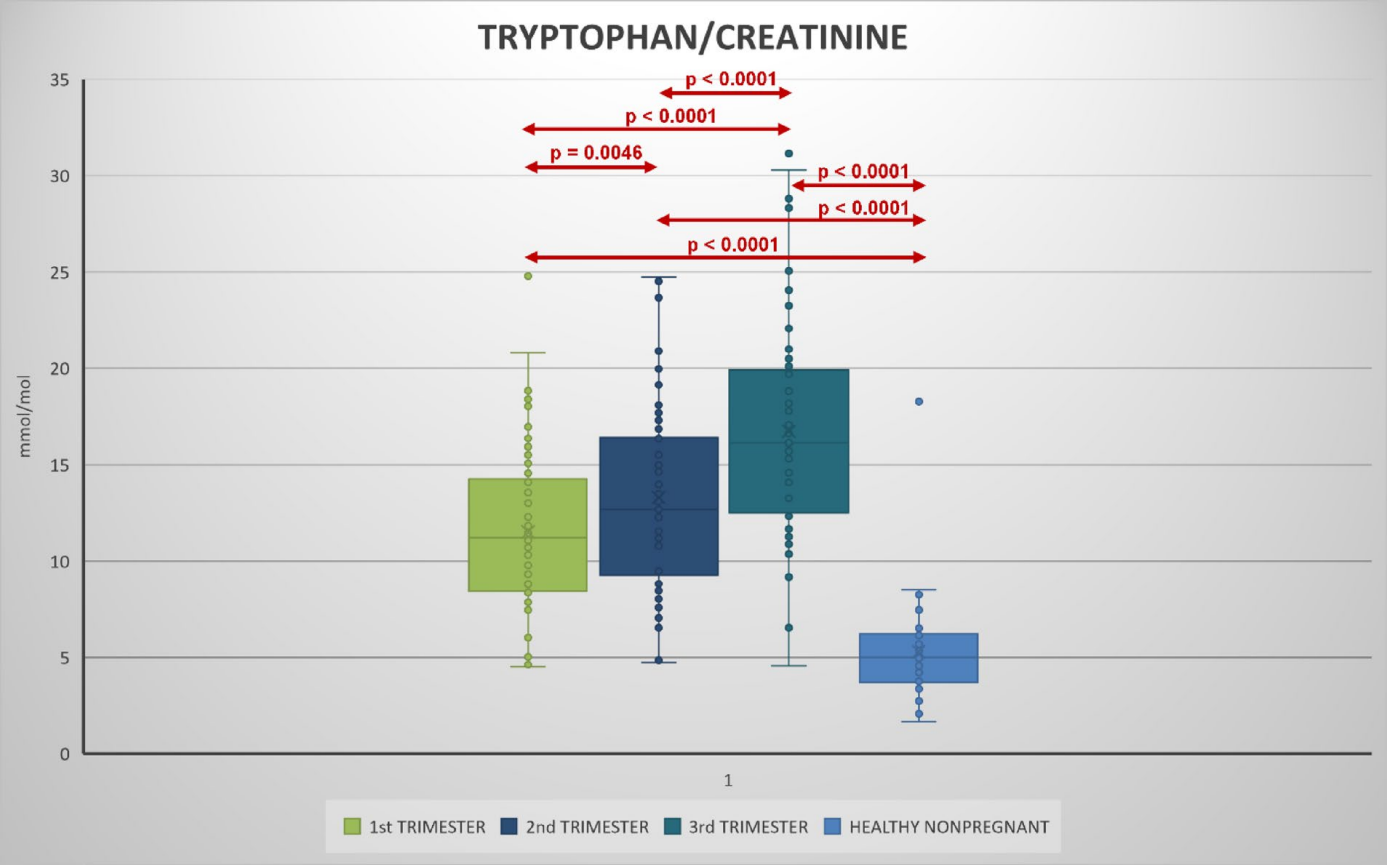
Urinary tryptophan/creatinine levels in 1st, 2nd, and 3rd trimester of pregnancy and its comparison during pregnancy and with healthy non-pregnant women.

Urinary kynurenine, tryptophan, kynurenine/tryptophan ratio increased with each trimester (Figs. 2, 3, 4, 5, Table S2).

**Fig. 6:**
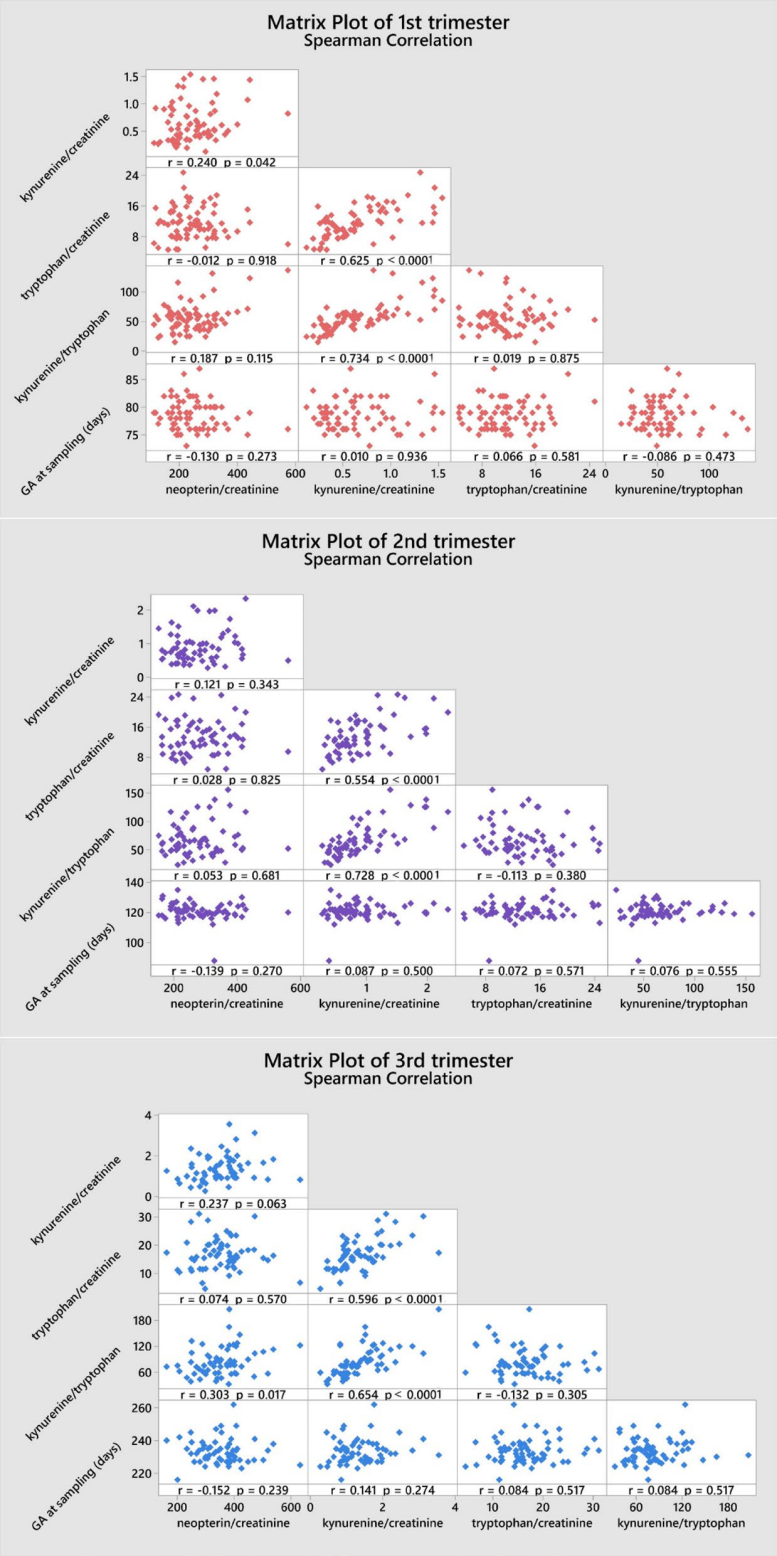
Spearman Correlation of neopterin, kynurenine, and tryptophan with gestation age and among these parameters in healthy pregnant women.

**Fig. 7:**
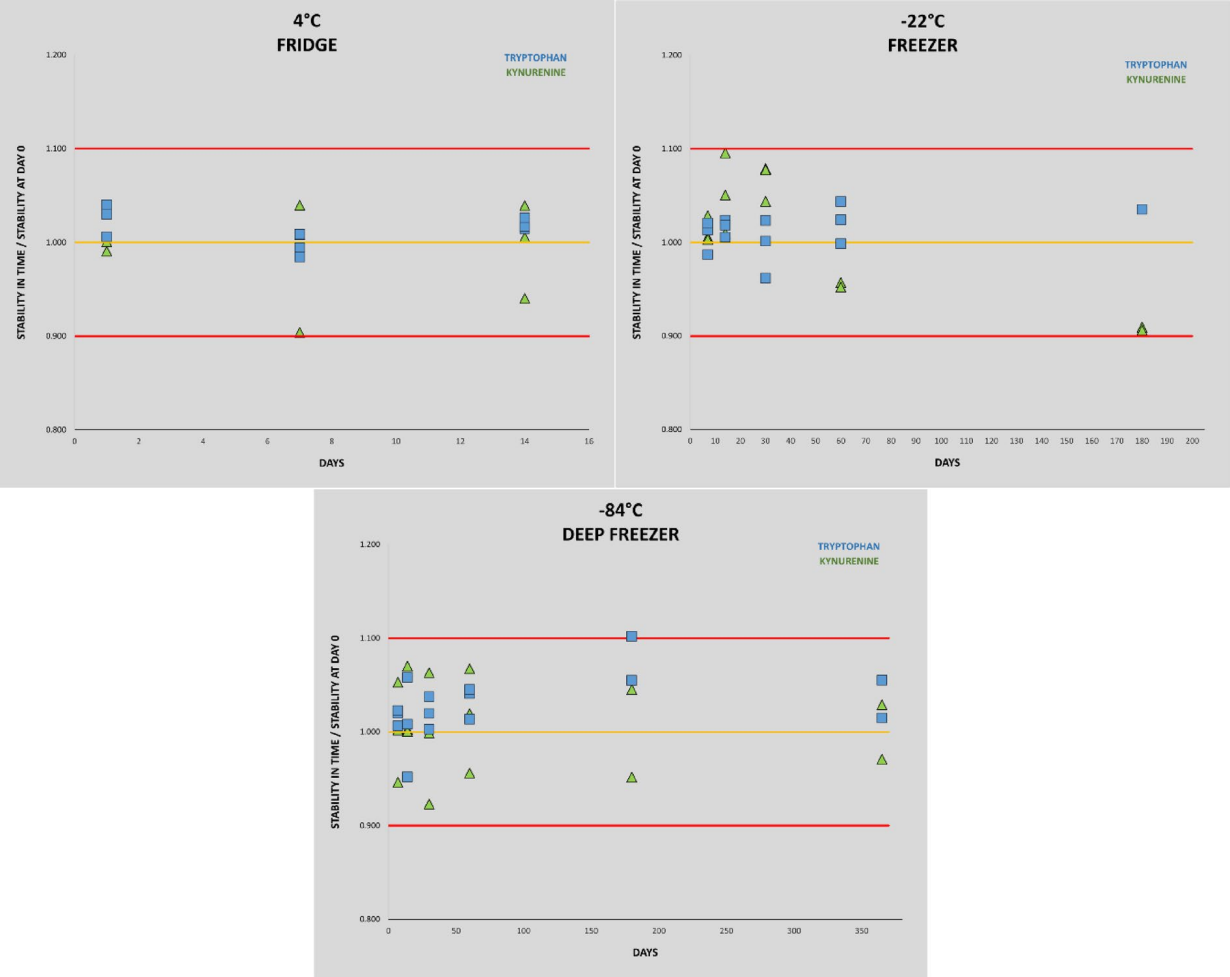
Stability of kynurenine and tryptophan in urine at different storage conditions.

Compared to the non-pregnant women neopterin, kynurenine and tryptophan concentrations in the 1st and 2nd trimester and neopterin, kynurenine, tryptophan concentrations and kynurenine/tryptophan ratio in the 2nd and 3rd trimester were significantly higher. Statistically significant lower concentrations in the non-pregnant women in comparison with healthy pregnant women in 1st, 2nd trimester in neopterin, kynurenine and tryptophan, and 3rd trimester in neopterin, kynurenine, tryptophan, and the kynurenine/tryptophan ratio were observed. The kynurenine/tryptophan ratio in the 1st trimester was similar to the non-pregnant women. No significant correlation was observed between urinary neopterin, kynurenine, tryptophan and gestational age or between the studied biomarkers (with the exception of an expected correlation between kynurenine and tryptophan) of different women within each trimester (Fig. 6). The stability of urine samples for the measurement of kynurenine and tryptophan was investigated under different storage conditions. Kynurenine and tryptophan in urine samples are stable for 14 days in the refrigerator at 4 °C, 6 months in the freezer at − 22 °C, and 12 months in the deep freezer at − 84 °C. Sample extracts can also be stored in autosampler at 4 °C for 24 h. Stability charts are presented in Fig. 7.

## Discussion

To the best of our knowledge this is the first study investigating concentrations of kynurenine and tryptophan during pregnancy in urine. Neopterin concentrations have been determined simultaneously, and values of urinary neopterin concentrations observed are in agreement with prior reports^33^. Compared with a cohort of healthy non-pregnant women all studied biomarkers, including tryptophan, were generally increased throughout the course of pregnancy. A consistent gradual increase in urinary neopterin, kynurenine, tryptophan and kynurenine/tryptophan ratio was also evident in comparison between pregnancy trimesters, with the highest values observed in the third trimester. An increase in urinary neopterin and circulating serum kynurenine and kynurenine/tryptophan ratio has been previously documented, but tryptophan was shown to decrease in the blood during pregnancy^34,35^, while present results in urine show increased tryptophan concentrations. This apparent discrepancy could be explained by higher excretion of amino acids in pregnancy, including increased urinary tryptophan loss^36^. Moreover, while the levels of analytes in the blood are expressed as absolute concentrations, the ratio to creatinine is used in the urine. However, the kynurenine/creatinine ratio which is of importance in assessing the immune response increased consistently. Despite the fact that neopterin, kynurenine, and tryptophan levels correlate in plasma or serum in pregnancy, no correlation between the studied biomarkers in each trimester was observed. Kynurenine usually shows a negative correlation with tryptophan in blood, which reflects tryptophan metabolism. However, a positive correlation has been shown in urine, which may again be related to the higher urinary losses of tryptophan in pregnancy.

The interesting finding of this study is that there was no correlation observed between the studied parameters and gestational age in different women evaluated during the same trimester. This fact could be explained by large interindividual variability of the investigated biomarkers and limited time span of sample collection in each trimester of pregnancy.

An integral part of the present study was also to determine the long-term storage stability of urine samples prior to analysis of kynurenine and tryptophan.

To the best of our knowledge, long-term stability study of urinary kynurenine and tryptophan has not been published to date. There are only two studies dealing with urine samples stability for kynurenine/tryptophan, but both are dealing with short -term stability (72 and 168 h, respectively)^37,38^. Stability of kynurenine in different matrices were presented in review Mrstna et al. in 2023^16^.

Present stability study results may help in planning the logistics of large clinical trials involving a high number of samples. Short term stability is also advantageous for long sample sequences and for possible repetition of some analyses without the need for additional sample preparation. Long-term stability is particularly profitable for large clinical studies where samples are stored until complete collection. It is also beneficial for storing backup samples for possible repeat analysis. In our case, the study at − 84 °C was terminated after one year, but not because of samples instability, so we assume that the samples will be more stable for longer time, but this needs to be verified.

Neopterin stability at different conditions was reported in the review^39^. Neopterin in urine is stable at − 20 °C for several months. More detailed stability was done using Bonobo’s and Chimpanzees urine in study^40^ where was reported that urinary neopterin levels at the beginning and after 2 years of storage at −20 °C showed no significant differences. In conclusion, the present results demonstrate differences in urinary concentrations of kynurenine, tryptophan and neopterin between women with physiological pregnancy and healthy women. Simultaneous determination of kynurenine, tryptophan and neopterin should be explored in the assessment of disorders of pregnancy.

## Electronic supplementary material

Below is the link to the electronic supplementary material.

Supplementary Material 1.

## Data Availability

The datasets generated during and/or analysed during the current study are available from the corresponding author on reasonable request Raw and detailed data were publish using Zenodo 10.5281/zenodo.15094968.
