# Supplementary material for: Urinary kynurenine,tryptophan, and neopterin concentrations during physiological pregnancy
Source: Sci Rep. 2025 Jun 4;15:19674. doi: 10.1038/s41598-025-04642-9 (PMC12137588; doi:10.1038/s41598-025-04642-9)
Supplement: Supplementary file 1 — Supplementary Material 1. [file 41598_2025_4642_MOESM1_ESM.docx]

**Urinary kynurenine, tryptophan, and neopterin concentrations during physiological pregnancy**

Lenka Kujovská Krčmová^*^, Kateřina Matoušová, Lenka Javorská, Kristýna Mrštná, Nikola  Přívratská, Chaweewan Suwanvecho, Dorota Turoňová, Mohamed Farrag, Marian Kacerovský, Bohuslav Melichar

Table S1 Optimized multiple-reaction monitoring parameters for kynurenine, tryptophan and L-tryptophan-D5 (internal standard)

| analyte | Precursor ions (m/z) | Product ions (m/z) | Dwell time (ms) | Q1 (V) | CE | Q3 (V) |
| --- | --- | --- | --- | --- | --- | --- |
| kynurenine | 208.80 | 94.00 | 100 | −10.0 | −15.0 | −10.0 |
|  | 208.80 | 146.00 | 100 | −14.0 | −22.0 | −16.0 |
|  | 209.10 | 94.05 | 100 | −15.0 | −14.0 | −20.0 |
| tryptophan | 204.90 | 117.90 | 100 | −10.0 | −24.0 | −27.0 |
|  | 205.10 | 188.20 | 100 | −15.0 | −11.0 | −22.0 |
|  | 205.10 | 118.05 | 100 | −11.0 | −26.0 | −24.0 |
| L-tryptophan-D5 | 210.05 | 191.80 | 100 | −14.0 | −12.0 | −14.0 |
|  | 210.05 | 149.95 | 100 | −30.0 | −18.0 | −20.0 |
|  | 209.95 | 192.15 | 100 | −11.0 | −11.0 | −14.0 |

Table S2 Concentration of urinary neopterin, kynurenine, tryptophan, and kynurenine/tryptophan ratio in 1^st^, 2^nd^, and 3^rd^ trimester and its comparison during pregnancy and with healthy nonpregnant women

|  | **Concentrations of urinary neopterin kynurenine and tryptophan during pregnancy**  median/mean (range) | | | **Comparison of neopterin, kynurenine, and tryptophan levels between trimesters in healthy pregnancy** | | | **Comparison of neopterin, kynurenine, and tryptophan levels in healthy nonpregnant and healthy pregnancy women** median/mean (range) | | | |
| --- | --- | --- | --- | --- | --- | --- | --- | --- | --- | --- |
|  | 1^st^ trimester (n=73) | 2^nd^ trimester (n=65) | 3^rd^ trimester (n=62) | p 2^nd^ vs 1^st^  trimester | p 3^rd^ vs 1^st^  trimester | p 3^nd^ vs 2^nd^  trimester | nonpregnant controls (n=42) | p control vs 1^st^  trimester | p control vs 2^nd^  trimester | p control vs 3^rd^  trimester |
| **neopterin/creatinine ratio**  (µmol/mol) | 237/248  (114 – 574) | 265/281  (152 – 561) | 353/353  (163 – 632) | **< 0.0001** | **< 0.0001** | **< 0.0001** | 164/167  (53 - 328) | **< 0.0001** | **< 0.0001** | **< 0.0001** |
| **kynurenine/creatinine ratio**  (mmol/mol) | 0.54/0.65  (0.12 – 1.54) | 0.80/0.91  (0.27 – 2.34) | 1.24/1.37  (0.27 – 3.55) | **< 0.0001** | **< 0.0001** | **< 0.0001** | 0.24/0.34  (0.05-2.50) | **< 0.0001** | **< 0.0001** | **< 0.0001** |
| **tryptophan/creatinine ratio**  (mmol/mol) | 11.2/11.5  (4.5 – 24.8) | 12.7/13.3  (4.7 – 24.7) | 16.1/16.7  (4.6 – 31.1) | **0.0046** | **< 0.0001** | **< 0.0001** | 5.0/5.3  (1.7 - 18.2) | **< 0.0001** | **< 0.0001** | **< 0.0001** |
| **kynurenine/tryptophan ratio**  (mmol/mol) | 53.8/56.2  (14.0 – 136.7) | 60.0/68.1  (23.0 - 156.1) | 76.5/83.3  (32.2 – 205.8) | **0.0002** | **< 0.0001** | **< 0.0001** | 53.0/62.8  (12.4 - 440.8) | 0.8325 | **0.0238** | **< 0.0001** |

Bold value means statistically significant result (p≤0.05).
